# Fine-Scale Variation in Vector Host Use and Force of Infection Drive Localized Patterns of West Nile Virus Transmission

**DOI:** 10.1371/journal.pone.0023767

**Published:** 2011-08-19

**Authors:** Gabriel L. Hamer, Luis F. Chaves, Tavis K. Anderson, Uriel D. Kitron, Jeffrey D. Brawn, Marilyn O. Ruiz, Scott R. Loss, Edward D. Walker, Tony L. Goldberg

**Affiliations:** 1 Department of Pathobiological Sciences, University of Wisconsin, Madison, Wisconsin, United States of America; 2 Department of Environmental Studies, Emory University, Atlanta, Georgia, United States of America; 3 Programa de Investigación en Enfermedades Tropicales, Escuela de Medicina Veterinaria, Universidad Nacional, Heredia, Costa Rica; 4 Graduate School of Environmental Science & Global Center of Excellence Program on Integrated Field and Environmental Studies, Hokkaido University, Sapporo, Hokkaido, Japan; 5 Department of Natural Resources and Environmental Sciences, University of Illinois, Urbana, Illinois, United States of America; 6 Department of Pathobiology, University of Illinois, Urbana, Illinois, United States of America; 7 Conservation Biology, University of Minnesota, St. Paul, Minnesota, United States of America; 8 Department of Microbiology and Molecular Genetics, Michigan State University, Lansing, Michigan, United States of America; Institut Pasteur, France

## Abstract

The influence of host diversity on multi-host pathogen transmission and persistence can be confounded by the large number of species and biological interactions that can characterize many transmission systems. For vector-borne pathogens, the composition of host communities has been hypothesized to affect transmission; however, the specific characteristics of host communities that affect transmission remain largely unknown. We tested the hypothesis that vector host use and force of infection (i.e., the summed number of infectious mosquitoes resulting from feeding upon each vertebrate host within a community of hosts), and not simply host diversity or richness, determine local infection rates of West Nile virus (WNV) in mosquito vectors. In suburban Chicago, Illinois, USA, we estimated community force of infection for West Nile virus using data on *Culex pipiens* mosquito host selection and WNV vertebrate reservoir competence for each host species in multiple residential and semi-natural study sites. We found host community force of infection interacted with avian diversity to influence WNV infection in *Culex* mosquitoes across the study area. Two avian species, the American robin (*Turdus migratorius*) and the house sparrow (*Passer domesticus*), produced 95.8% of the infectious *Cx. pipiens* mosquitoes and showed a significant positive association with WNV infection in *Culex* spp. mosquitoes. Therefore, indices of community structure, such as species diversity or richness, may not be reliable indicators of transmission risk at fine spatial scales in vector-borne disease systems. Rather, robust assessment of local transmission risk should incorporate heterogeneity in vector host feeding and variation in vertebrate reservoir competence at the spatial scale of vector-host interaction.

## Introduction

Host community composition can exert a strong effect on vector-borne pathogen transmission when vertebrate reservoir competence varies among host community members [1], [2], [3], [4], [5]. Early studies of the relationship between host community structure and human disease [6] led to the proposal of ‘zooprophylaxis,’ where co-occurring vertebrate species may diminish the risk of vector transmitted diseases for a focal species, particularly humans [7], [8]. Mechanistically, the process involves the ‘diversion’ or ‘wasting’ of vector feeding effort towards less-competent hosts and away from humans or more-competent hosts [2], [9], [10]. Recently, this argument has been recast as a “dilution effect” [3], whereby host diversity itself reduces the risk of disease transmission. The appeal of “zooprophylaxis” or the “dilution effect” as a general principle derives from its focus on biodiversity as a barrier to vector-borne zoonotic disease transmission [11], [12], [13]. However, considering only diversity or richness as a measure of host community structure ignores ecological complexities inherent to any host-vector system, such as heterogeneities in vector host selection and variation in vertebrate reservoir competence.

The introduction and establishment of West Nile virus (WNV) into North America [14] offers an opportunity to explore associations between host community composition and arbovirus transmission. WNV is maintained in an enzootic transmission cycle by *Culex* spp. mosquitoes, principally *Culex pipiens* in the eastern United States north of 36° latitude [15], [16], [17], and a suite of bird species that vary in their competence [18], [19]. Previous studies have reported inverse associations between non-passerine bird species richness and human WNV cases [20], and between WNV infection in *Culex* mosquitoes and the percent of wetland cover [21], as well as positive associations between high avian diversity and low WNV incidence in humans in the eastern United States [22]. Allan et al [23] found that WNV infection in mosquitoes and incidence in humans increased with decreasing bird diversity and increasing vertebrate reservoir competence of the bird community, while Koenig et al [24] found that the decline of the American crow (*Corvus brachyrhynchos*) was accelerated in areas of low avian diversity. Although all of these studies suggest a pattern in which increased host diversity or richness dampens WNV transmission, these studies have not accounted for host selection by vectors, a potentially critical determinant of pathogen transmission, given that vectors do not feed in proportion to host abundance [16], [25], [26], [27].

Selective feeding by vectors in the case of WNV suggests that a small number of avian species (i.e. ‘super-spreaders’) might be responsible for the majority of WNV transmission [16], [28], even when avian community diversity is high. Further, non-random host selection by mosquitoes modifies the effect of vertebrate reservoir competence on the prevalence of WNV in vectors [19], [25]. Described as heterogeneities in the host community, non-random host selection has been observed in other disease systems [29], [30], [31], with results suggesting nonlinear effects on pathogen transmission. The processes of host-selection, and more broadly host-vector contact, operate on a very fine spatial scale; therefore, analysis of these relationships at coarser scales (e.g. county or region) could easily obscure important local patterns that affect pathogen transmission more directly [32].

To test the hypothesis that attributes of the host community such as vertebrate reservoir competence and selection by arthropod vectors may be more accurate predictors of arboviral transmission than vertebrate host diversity or richness, we characterize the WNV transmission cycle at a fine spatial scale within an urban focus of infection in Chicago, USA. In particular, we focus on “host community force of infection”, defined here as the summed number of infectious mosquitoes resulting from each vertebrate host upon which vectors feed. This quantity incorporates empirical measures of mosquito host selection derived from blood meal analyses of mosquitoes and indices of vertebrate reservoir competence. We focus on *Culex* mosquitoes, the primary vectors of WNV in North America, because host selection is known to vary across North America for this vector [26], [28]. We compare fine-scale *Culex* feeding patterns in relation to bird communities surveyed at the same sites. Finally, we model several characteristics of the host community, including community force of infection, avian diversity, and richness as predictors of WNV infection in *Culex* mosquitoes.

## Materials and Methods

### Study area and sampling

The study region in southwest Chicago, Illinois (Cook County; 87°44′ W, 41° 42′ N) consisted of 26 different residential sites and five “semi-natural sites” (three cemeteries, one wildlife refuge, and one forest preserve). Permission to conduct this research was obtained from the Villages of Alsip, Evergreen Park, Indian Head Park, Oak Lawn, Palos Hills, Western Springs, the City of Blue Island, Burbank, Chicago, Harvey, the Archdiocese of Chicago, and many private homeowners within these municipalities. Residential sites were selected to represent a range of human population densities and distances to semi-natural areas, as previously described [33], [34]. To estimate mosquito abundance and infection, we deployed CDC light and gravid traps from mid-May to mid-October in 2005–2008; after species identification, we pooled individuals according to species, location of collection, and blood-feeding status (fed or non-fed; details in Methods S1). We used quantitative real-time polymerase chain reaction (qRT-PCR) to detect WNV and estimated the annual per-site *Culex* spp. mosquito infection rate according to the maximum likelihood method [35]. In addition, we estimated the relative abundance of *Culex* spp. mosquitoes (number per light trap night) to control for vector density effects which are known to influence relationships between host communities and disease risk [36]. We used the blood-fed *Culex pipiens* for molecular identification of vertebrate blood meal source (see Methods S1). We surveyed bird communities in 2006 using point counts [34] and estimated bird densities using the program Distance 5.0 [37].

#### Host selection

Host feeding selection for birds was estimated using the Manly resource selection design II index [38], a ratio that uses relative density as the measure of host availability (density-based selection ratio; 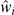) and was estimated for *Cx. pipiens* at follows:
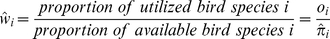
The Manly selection ratio equals 1 when mosquito feeding on host *i* is in equal proportion to estimated availability; is >1 when a host is overused (*i.e.* more frequent feeding than expected by chance), and is <1 when a host is underused (*i.e.* less frequent feeding than expected by chance). The selection index and standard error were calculated using the adehabitat package in Program R [39]. We collected *Culex pipiens* blood-feeding data at 23 study sites that also had bird survey data, for the purpose of this study, and in order to maintain statistical power, we present results for sites with at least 18 avian-derived blood meals (n = 11 sites). Over- or under-utilization of a host species was considered statistically significant when the 95% confidence interval did not overlap unity.

When estimating the Manly host selection ratio, bird species that were not observed as blood meal hosts but were identified in bird surveys were given a blood meal value of one. Bird species observed as blood meal hosts but not observed in bird surveys were given a density equal to the lowest observed bird density at each site. Host selection values were aggregated by site across years, since sample sizes in some years at some sites were too low for meaningful statistical analysis.

### Force of infection

For each of the sites, we estimated the number of infectious *Cx. pipiens* mosquitoes (*F_i_*) resulting from vector feeding on each host according to F_i_  =  B_i_^2^ * C_i_ [26], where *B_i_* equals the fraction of the total blood meals from host *i* and *C_i_* equals the vertebrate reservoir competence index [18]. Bird species without a competence index were assigned the average competence value for their respective taxonomic family [18]. For several species, family-level competence values were not available, so the average competence for the respective avian order was assigned (Passeriform  =  0.773; Charadriiform  =  1.018). The competence for all mammalian hosts was zero except for gray squirrel (*Sciurus carolinensis*) (*C_i_* = 0.066; [40]). The force of infection assumes equal initial seroprevalence among hosts and equal feeding rates and competence values for adult and juvenile birds.

To characterize community-wide force of infection, we used the sum of *F_i_* for each study site. Originally, force of infection described the *per capita* rate at which a susceptible individual acquires infection [41]. In a system with multiple species of hosts, the expression represents the total force of infection exerted all host species in a community [1]. We report community force of infection for sites with at least 20 identified *Cx. pipiens* blood meals (data aggregated among years) but for the modeling described below, we calculated community force of infection for sites within years.

### Statistical analysis

To explore associations between *Cx. pipiens* blood feeding patterns and local avian diversity measures, we compared diversity index values and richness for blood meal data and bird survey data at the same sites. Estimated species diversity was derived using the Shannon index, which incorporates both species richness and evenness [42]. In calculating the diversity of blood meal data, we aggregated data across 4 years to maintain statistical power and only included 16 sites in the analysis that had at least 10 identified avian *Cx. pipiens* blood meals (493 blood meals distributed among 16 sites). We used paired *t*-tests to evaluate the associations of avian diversity and richness and blood meal diversity and richness among the sites.

For the initial modeling effort, we used linear mixed effects models to examine relationships among *Culex* spp. infection rate, attributes of the host community (community force of infection, avian diversity, avian richness), and vector abundance (number of *Culex* spp. per light trap night). Sites and years were included as random factors and the significance of the fixed factors was estimated with a parametric bootstrap [43]. We included weights to account for unequal variance and unequal numbers of observations in estimating the fraction of total blood meals from host *i*. Specifically, weights were proportional to the number of blood-fed mosquitoes collected among sites. Candidate models included all combinations of these variables, including single-variable models [44].

For the subsequent modeling effort, we used general linear models to investigate the relationship between force of infection for each bird species (*B_i_* ^2^ * *C_i_*) and *Culex* infection rate. This second modeling effort did not include site and year as random effects because previously fitted linear mixed effects models revealed those factors to have zero variance. Candidate models included force of infection for avian species that were abundant or commonly fed upon [(American robin (*Turdus migratorius*), house finch (*Carpodacus mexicanus*), house sparrow (*Passer domesticus*), mourning dove (*Zenaida macroura*), northern cardinal (*Cardinalis cardinalis*), and European starling (*Sturnus vulgaris*)]. To determine the sensitivity of sample size on the results of the models, we created separate statistical models using data for sites with at least 8, 10, and 15 identified *Cx. pipiens* blood meals per year (resulting in sample sizes of 27, 21, 14, respectively). The results of the models for the different datasets with different sample size cut-offs were similar and results are presented for the cut-off of 10. We used the Akaike Information Criterion with a bias correction term for small samples size (AICc) to evaluate candidate models [44]. Residuals from models were inspected with diagnostic plots to ensure that model assumptions were met (see Figure S1). All tests were computed in R v2.11.1 statistical programming language [39].

## Results

### Mosquito collection

**Figure 1:**
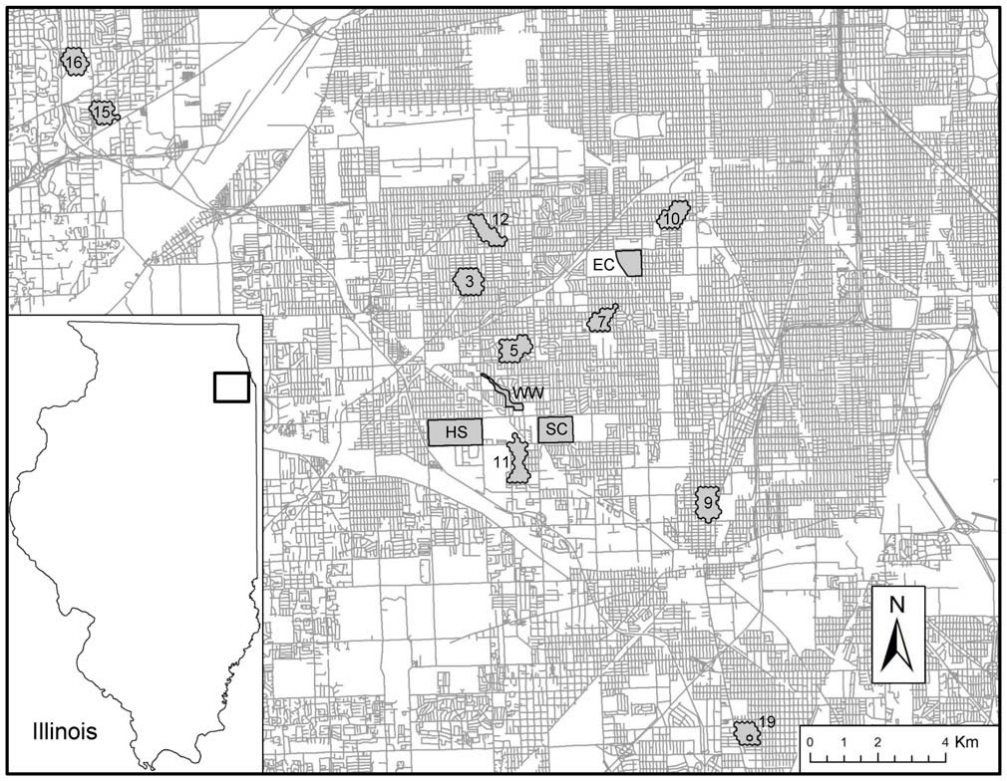
Map of study sites in suburban Chicago, Illinois, U.S.A. Site names are as follows: Oak Lawn - North (3), Oak Lawn - Central (5), Evergreen Park - West (7), Blue Island (9), Chicago - Ashburn East (10), Alsip (11), Burbank (12), Indian Head Park (15), Western Springs (16), Harvey (19), Holy Sepulchre Cemetery (HS), Saint Casimir's Cemetery (SC), Evergreen Cemetery (EC), Wolfe Wildlife Refuge (WW).

Between 2005 and 2008, we collected 2,971 *Culex* spp. pools, totaling 57,053 individuals. Infection rates for each year and site ranged from 9.8 per 1,000 (C.I.  = 5.2–17.1) to 30.7 per 1,000 (C.I.  =  17.0–52.9) for the 14 sites with host community force of infection data (Fig. 1). The average number of *Culex* spp., mosquitoes captured per light trap night within years (early-Jul. to late-Sept.) ranged from 0.8 to 60.3 (mean of 16.4 and median of 10.8).

### *Culex pipiens* host selection, avian community structure, and force of infection

We collected 1,614 blood-fed mosquitoes, and of the *Culex* spp. mosquitoes collected, 869 were *Cx. pipiens* (64.9%), 256 were *Cx. restuans* (19.1%), 2 were *Cx. salinarius* (0.1%). A total of 213 *Culex* mosquitoes (15.9%) could not be identified using our PCR-based methods. We obtained blood meal identifications for 1,085 of the total individuals (67.2%) and 652 of the *Cx. pipiens* (75.0%). The over- or under-utilization of each host species by *Cx. pipiens* was not consistent among sites (Table S1).

We found that the diversity of hosts fed upon by *Cx. pipiens*, as determined by blood meal analysis, was significantly different from avian diversity at the same site (t = −2.78, d.f.  =  15, *P* = 0.014) and that blood meal richness was significantly different from avian richness (t = −5.73, d.f.  =  15, *P*<0.001). Sites with high avian diversity based on point count surveys did not have high diversity of birds that were fed upon by *Cx. pipiens*. Birds surveyed at high diversity sites such as SC and WW (both urban green spaces) that were not represented in the 652 blood meals from the study region included American crow, brown-headed cowbird (*Molothrus ater*), black-crowned night-heron (*Nycticorax nycticorax*), Canada goose (*Branta canadensis*), common yellowthroat (*Geothlypis trichas*), downy woodpecker (*Picoides pubescens*), eastern kingbird (*Tyrannus tyrannus*), eastern meadowlark (*Sturnella magna*), great blue heron (*Ardea herodias*), mallard (*Anas platyrhynchos*), monk parakeet (*Myiopsitta monachus*), red-eyed vireo (*Vireo olivaceus*), ring-necked pheasant (*Phasianus colchicus*), warbling vireo (*Vireo gilvus*), willow flycatcher (*Empidonax traillii*), and yellow warbler (*Dendroica petechia*).

The aggregate force of infection for all sites and years combined demonstrated the American robin to rank highest, accounting for 86.7% of the total force of infection, followed by house sparrow (9.1%) and house finch (2.1%; Table S2).

### Modeling host community structure and *Culex* infection rate

**Figure 2:**
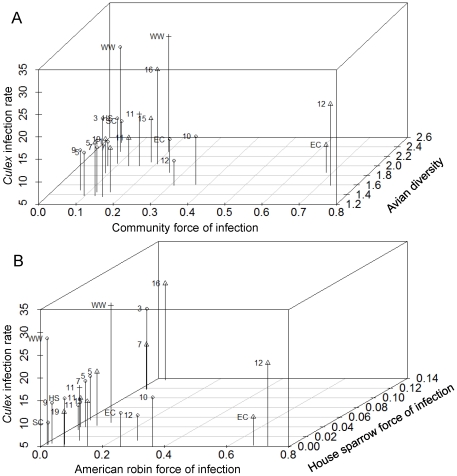
Scatterplots showing relationships between *Culex* spp. mosquito infection rate (# positive per 1,000) and host community force of infection and avian diversity (Shannon index) (A), and American robin and house sparrow force of infection (B), in study sites in southwest suburban Chicago, Illinois, U.S.A., 2005-2008. Symbols represent year (circle  =  2005, triangle  =  2006, cross  =  2007), and labels are the site identification code.

**Figure 3:**
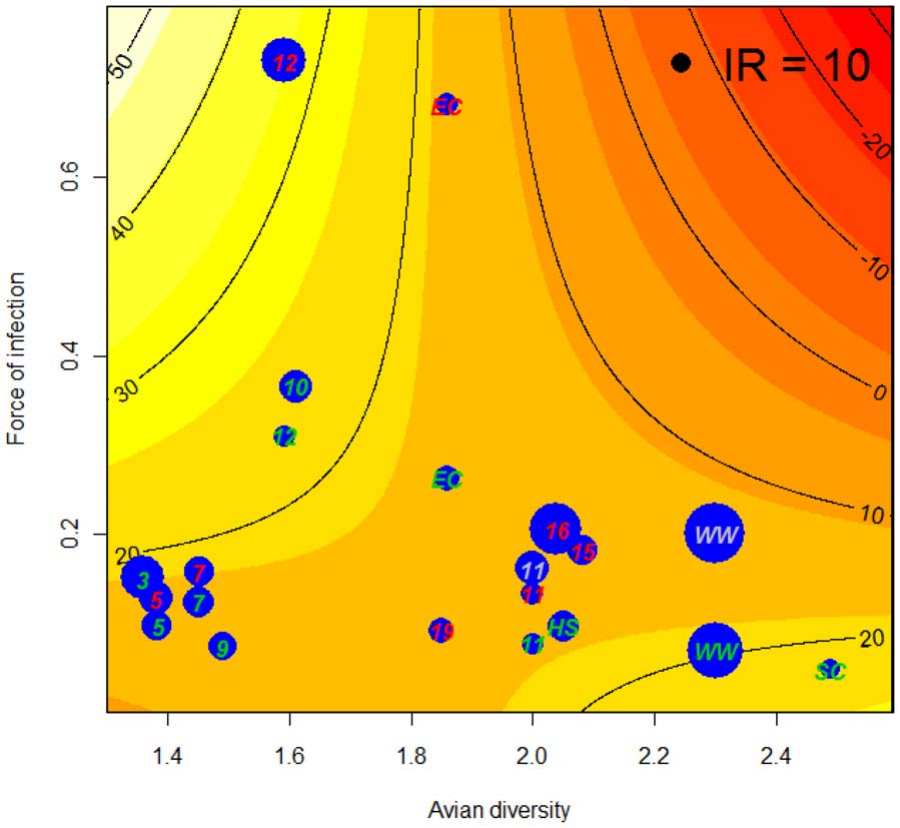
Model and data depicting the interaction between host community force of infection and avian diversity (Shannon index). Colors represent model expected values for the infection rate, IR (indicated by the contour lines); blue dots represent the IR estimates from the mosquito pools (dot size is proportional to IR magnitude, for reference the black dot on the top right corner represents an IR = 10). Site identification code is indicated on top of each IR estimate and color indicates the year of the estimate (green = 2005, red = 2006, gray = 2007).

Host community force of infection for each site ranged from 0.05 to 0.73 (mean of 0.21±0.04; Fig. 2A). To investigate the influence of host community structure on *Culex* infection rate, a model selection procedure indicated that the data were best fit by a model (lowest AICc value and highest weight) that included an interaction term of community force of infection and diversity (Fig. 3; Table 1; parameter ± S.E.  =  −101.63±3.35, 95% bootstrap confidence intervals  =  −127.05 to −75.10, *P* = 0.036). The main effects of this model were not significant (community force of infection parameter  =  188.58±5.48, *P* = 0.179; avian diversity parameter  =  12.65, *P* = 0.086).

**Table 1:** Candidate models for predicting *Culex* infection rate with site and year included as random effects in the linear mixed effect model with unequal variance.

Model	AICc	ΔAICc	w_i_
FOI*Div	181.2	0.0	0.5851
FOI+Div+FOI*Div	183.8	2.6	0.1604
FOI*Div+Rich	183.9	2.7	0.1526
FOI*Rich+Div	185.3	4.1	0.0758
FOI*Rich	188.3	7.1	0.0168
FOI*Div+Rich+CxDen	190.4	9.2	0.0059
FOI*Rich+Div+CxDen	192.5	11.3	0.0021
CxDen+FOI*Rich	195.2	14.0	0.0005
FOI+Div	195.6	14.4	0.0004
FOI	197.9	16.7	0.0001
FOI*CxDen+Div	198.2	17.0	0.0001
FOI*CxDen	200.6	19.4	0.0000
FOI*CxDen+Div+Rich	200.7	19.5	0.0000
FOI+Rich	201.6	20.4	0.0000
FOI+CxDen	202.9	21.7	0.0000
Div	203.7	22.5	0.0000
Rich+FOI*CxDen	203.9	22.7	0.0000
FOI+Div+Rich+CxDen	204.2	23.0	0.0000
Rich	208.9	27.7	0.0000
CxDen	210.5	29.3	0.0000

FOI  =  community force of infection; Div  =  avian diversity; Rich  =  avian richness; CxDen  =  *Culex* captured per light trap night.

We further explored the relationship between the force of infection (*B_i_* ^2^ * *C_i_*) for individual bird species and *Culex* infection rate. The linear models did not include random factors of year and site due to the lack of variation explained by those factors. A model selection process including all combinations of variables resulted in 54 competing models and the best model (lowest AICc) included the force of infection for American robin, house sparrow, and European starling (Table 2 and Fig. 2B; AICc  =  140.1, F = 3.27, r^2^ = 0.25, d.f.  =  3 and 17, and *P* = 0.047). The second best model explaining variation in *Culex* infection rate included American robin and house sparrow force of infection (AICc  =  140.5, F = 3.22, r^2^ = 0.18, d.f.  =  2 and 18, and *P* = 0.064).

**Table 2:** Model parameters for the top ranked model utilizing individual bird force of infection to predict the *Culex* infection rate in suburban Chicago, 2005-2007.

Variable	Estimate	S.E.	t-value	*P*-value
American robin force of infection	12.12	4.48	2.71	0.015
House sparrow force of infection	63.18	34.66	1.82	0.086
European starling force of infection	1797.98	1084.51	1.66	0.116

## Discussion

*Culex* infection rate was best explained by a model including the interaction of host community force of infection and host diversity. Specifically, the interaction of community force of infection and avian diversity yielded a significant nonlinear relationship as predictors of the prevalence of virus infection in the vector mosquito population. This interaction implies that increases in avian diversity do not result in a concomitant linear increase or decrease in *Culex* infection rate. Rather, the direction of change in the host diversity and infection interaction depends upon parallel changes in host selection and the reservoir competence of those hosts. Sites with moderate levels of avian diversity tended to have higher host community force of infection. This interaction is similar to the interaction of landscape features on mosquito species richness at the same study sites, where the most heterogeneous landscapes harbored the largest number of species [45]. Importantly, the models that included force of infection had higher explanatory power than models including only avian richness or diversity. Moreover, force of infection from two species, the American robin and the house sparrow, were significant predictors of WNV infection in *Culex* spp. mosquitoes.

When estimating the proportional contribution of each avian species to WNV transmission, our model incorporated birds utilized as hosts by arthropod vectors and did not rely on birds that were only surveyed during point counts. As a result, our model was able to account indirectly for several avian functional traits that lead to non-random host selection, thereby producing a metric of community force of infection that would not have been attainable otherwise. By indirectly modeling traits such as avian body mass, roosting habitat, and anti-mosquito behavior alongside mosquito functional traits such as host location [46], we were able to calculate a biologically realistic index of the risk of WNV transmission. In this sense, our analysis is similar to measures of “functional diversity" that describe organismal traits that influence ecosystem function and productivity [47], [48].

Our results show that certain members of the avian community have disproportionate contributions to amplification and transmission of WNV. This result, in turn, demonstrates that avian diversity and richness as measured by point counts are uncoupled from the diversity and richness of birds utilized as hosts by *Cx. pipiens* at the same sites. We also show that 16 avian species detected by bird surveys were not identified in *Cx. pipiens* blood meals from the same sites, despite extensive sampling. In a recent study, McKenzie and Goulet [49] considered non-random host selection and variation in vertebrate reservoir competence to WNV when evaluating the influence of the host community composition to WNV disease risk. Their study suggested that a small component of the bird community, characterized by species with high amplification fractions [16], were significant predictors of human WNV cases in Colorado, USA. Taken together, these results demonstrate that the mere presence of a species in a community of potential hosts does not necessarily merit its inclusion as a contributor to the force of infection of the vector population.

Our current and previous results [16] provide a spatially detailed description of *Cx. pipiens* host selection. When calculating host selection among sites at a fine spatial scale, we show that the over-utilization of robins is not always consistent among sites. For example, at site SC (Saint Casimir's cemetery), robins were significantly under-utilized, meaning that they were used less than would be expected based on availability, which is contrary to the selection index for robins observed in previous studies [16], [26], [28]. One explanation for this pattern is that the bird communities surveyed during the day do not necessarily reflect the roosting birds available to mosquitoes at night. During ongoing research, we observed that radio-tagged robins that are present in this cemetery during the day primarily roost outside the cemetery at a large communal roost, which implies that these robins are unavailable to night-time host-seeking mosquitoes in this cemetery.

Calculating the force of infection for individual species at each site, and for the study area as a whole identified American robins as having the highest force of infection (0.160), followed by house sparrow (0.017), house finch (0.004), and northern cardinal (0.002). These results highlight that American robin and house sparrow produced 95.8% of the infectious *Cx pipiens* mosquitoes and were positively associated with WNV infection in *Culex* mosquitoes. These results support the broad importance of American robins as WNV amplification hosts [16], [26], [28], and offer new data in support of the contribution of house sparrows and house finches to WNV transmission [26], [50].

One assumption of this study is that avian host behavior is not detrimental to mosquito survival. Mortality risk from predation by vertebrate hosts has been observed in the field [51], [52], and simulation modeling has demonstrated a trade-off between feeding persistence of mosquitoes [53] and the rate at which vectors die while searching for a blood meal [54]. A recent empirical study identified 9% mortality in *Cx. pipiens* mosquitoes that attempted to feed on house sparrows or chickens [55]. Keesing et al. [56] recently evaluated how host community composition could affect tick survival and found that some hosts can kill thousands of ticks per hectare. These hosts act as “ecological traps” [57], and resulted in an estimated 82.8% to 96.5% mortality in larval *Ixodes scapularis* ticks. This rate of host-related vector mortality is probably much greater in ticks than mosquitoes; however, more data evaluating interspecific variation in mosquito predation is warranted.

This study takes the novel approach of incorporating host community force of infection as a key variable for explaining fine scale variation in transmission of a multi-host mosquito-borne pathogen. We observed a relationship between *Culex* infection rate and the interaction of host community force of infection and avian diversity; this pattern was driven by virus amplification in two bird species, the American robin and house sparrow. Ideally, future studies would focus on providing insights into the processes that explain the relationships between host community force of infection and disease risk, and on the ultimate goal of informing a general model for WNV occurrence across spatial scales. Taken together, our results suggest that the influence of host community structure on vector-borne disease risk is conditional and influenced by heterogeneity in vector-host contact and variation in competence within the vertebrate reservoir community.

## Supporting Information

Methods S1 This file includes additional methodological descriptions regarding mosquito collection, laboratory diagnostics, and avian host surveys.
(DOC)

Figure S1 This file contains the diagnostic plots (residuals and Q-Q plot) for the statistical models.
(DOC)

Table S1 This file is a table of *Cx. pipiens* host selection ratios for each host species at 11 field sites in southwest suburban Chicago, Illinois.
(DOC)

Table S2 This file contains host species force of infection values which represent the number of infectious *Cx. pipiens* mosquitoes resulting from feeding on each host species.
(DOC)
