# Supplementary material for: Fine-Scale Variation in Vector Host Use and Force of Infection Drive Localized Patterns of West Nile Virus Transmission
Source: PLoS One. 2011 Aug 19;6(8):e23767. doi: 10.1371/journal.pone.0023767 (PMC3158794; doi:10.1371/journal.pone.0023767)
Supplement: Methods S1 — This file includes additional methodological descriptions regarding mosquito collection, laboratory diagnostics, and avian host surveys. (DOC) [file pone.0023767.s001.doc]

**SUPPORTING DOCUMENT**

**Methods S1** This file includes additional methodological descriptions regarding mosquito collection, laboratory diagnostics, and avian host surveys.

**Mosquito collection, identification, and WNV infection rate**

Mosquitoes were sampled from study sites once every two to three weeks from mid-May through mid-October in 2005—2008, using CO_2_-baited CDC miniature light traps, CDC gravid traps baited with rabbit pellet infusion, and battery-powered backpack aspirators. The number of light traps and gravid traps deployed at each site was consistent within each year. Mosquitoes were identified to species morphologically [1] and blood-fed individuals were separated from gravid and unfed individuals. *Cx. pipiens* and *Cx. restuans* were pooled together during processing due to the difficulty in distinguishing the two based on morphological characteristics [2]. Non-blood-fed female mosquitoes were pooled into groups of 50 or less, by species, location of collection, and tested for WNV RNA using reverse transcription, real time polymerase chain reaction (PCR). Mosquitoes were homogenized by adding 1 mL of a 50:50 mixture of phosphate-buffered saline (PBS) and 2X lysis buffer (Applied Biosystems, Foster City, CA) and three #7 steel shots using a high-speed mechanical homogenizer (Retsch MM 300) for 4 minutes at 20 cycles/second. Each homogenized pool was centrifuged for 2 minutes at 13,000 rpm. RNA was extracted from mosquito pools using an ABI Prism 6100 Nucleic Acid Prep Station following the Tissue RNA Isolation Protocol (Applied Biosystems; P/N 4330252). RNA was eluted in a final volume of 60 µL of elution solution. A region of the WNV RNA envelope gene was detected using real-time, reverse transcription polymerase chain reaction (RT-PCR) [3]. The thermocycling was performed on an ABI Prism 9700HT sequence detector at the Research Technology Support Facility at Michigan State University, following the Taq- Man One-Step RT-PCR Master Mix Protocol (Applied Biosystems; P/N 04310299). The amplification threshold was determined by serial dilutions of controls and a maximum cycle threshold for samples to be considered positive was 40. Mosquitoes were processed during the RNA extraction and RT-PCR on 96-well plates and each plate contained at least two positive extraction controls and an additional RT-PCR positive control. All plates had at least 10 negative controls (blanks) distributed evenly on the plate for quality control. Results were only accepted when positive and negative controls on each plate were clean and all positive field collected pools were confirmed by a second extraction and RT-PCR. Maximum likelihood estimates for infection rates were calculated using the Pooled Infection Rate version 3.0 Microsoft Excel add-in [4].

Spatial variation in disease risk at each site was estimated as the mean *Culex* spp. infection rate among years. Spatial variation in vector density was estimated as the number of *Culex* spp. mosquitoes captured per light trap night for each year. Only data from weeks 27 to 38 (early July to late September) were used to calculate *Culex* infection rates and *Culex* relative abundances; in doing so, trap sampling effort was standardized among years. Blood-fed *Culex* spp. mosquitoes were identified to species using a PCR based method and the remainder of the mosquito was tested for WNV.

**Mosquito blood meal analysis**

Following the methods described by Hamer et al. [5], DNA was extracted from the blood-fed abdomen of mosquitoes and a series of PCRs were used to amplify different regions of the cytochrome b gene of putative blood meal sources. PCR amplicons were visualized, purified, and sequenced directly on ABI 3730 Genetic Analyzer at the Research Technology Support Facility at Michigan State University. Sequences were queried against GenBank using the BLAST algorithm to identify the closest match, which was assigned as the blood meal. Negative controls were carried through all steps, and 16 species of birds, 8 species of mammals, and 2 species of amphibians were correctly identified as positive controls.

**Host community surveys**

Local bird abundance was estimated at each site in 2006 using the point count method [6,7]. Five points were established in each of the twenty-one residential sites and eight points in each of the five natural sites. Surveys were conducted between 0.5 hour before sunrise and 4.0 hours after sunrise (0530-1000) on days with no precipitation and wind speed less than 24 km/hr. Two surveys were conducted between June and mid-July, corresponding to the peak of avian breeding in the region. Five-minute unlimited radius point counts were conducted at each survey point, distance to each observed bird was recorded, and density of each species and total avian density were estimated using Program DISTANCE 5.0 [8]. Relative abundance of individual species was calculated by dividing species density by total bird density.

**References**

1. Andreadis TG, Thomas MC, Shepard JJ (2005) Identification Guide to the Mosquitoes of Connecticut. New Haven, CT: The Connecticut Agricultural Experiment Station.

2. Harrington LC, Poulson RL (2008) Considerations for accurate identification of adult Culex restuans (Diptera : Culicidae) in field studies. J Med Entomol 45: 1-8.

3. Lanciotti RS, Kerst AJ, Nasci RS, Godsey MS, Mitchell CJ, et al. (2000) Rapid detection of West Nile virus from human clinical specimens, field-collected mosquitoes, and avian samples by a TaqMan reverse transcriptase-PCR assay. J Clin Microbiol 38: 4066-4071.

4. Biggerstaff BJ (2006) PooledInfRate, Version 3.0: a Microsoft Excel Add-In to compute prevalence estimates from pooled samples. Centers for Disease Control and Prevention. Fort Collins, CO.

5. Hamer GL, Kitron UD, Goldberg TL, Brawn JD, Loss SR, et al. (2009) Host selection by Culex pipiens mosquitoes and West Nile virus amplification. Am J Trop Med Hyg 80: 268-278.

6. Hutto RL, Pletschet SM, Hendricks P (1986) A fixed-radius point count method for nonbreeding and breeding-season use. Auk 103: 593-602.

7. Loss SR, Hamer GL, Walker ED, Ruiz MO, Goldberg TL, et al. (2009) Avian host community structure and prevalence of West Nile virus in Chicago, Illinois. Oecologia 159: 415-424.

8. Thomas L, Buckland ST, Rexstad EA, Laake JL, Strindberg S, et al. (2010) Distance software: design and analysis of distance sampling surveys for estimating population size. J Appl Ecol 47: 5-14.
