# Supplementary material for: Fine-Scale Variation in Vector Host Use and Force of Infection Drive Localized Patterns of West Nile Virus Transmission
Source: PLoS One. 2011 Aug 19;6(8):e23767. doi: 10.1371/journal.pone.0023767 (PMC3158794; doi:10.1371/journal.pone.0023767)
Supplement: Figure S1 — This file contains the diagnostic plots (residuals and Q-Q plot) for the statistical models. (DOC) [file pone.0023767.s002.doc]

SUPPORTING DOCUMENT

**Figure S1 Diagnostic plots for models explaining variation in *Culex* infection rate.** Part A are the residuals for the model including the interaction of community force of infection and diversity (FOI*Div; see Table 1) and part B are the residuals for the model containing the individual force of infection for American robin, house sparrow, and European starling (see Table 2). Symbols in the residual plot indicate site and color indicates year.
