# Supplementary material for: Fine-Scale Variation in Vector Host Use and Force of Infection Drive Localized Patterns of West Nile Virus Transmission
Source: PLoS One. 2011 Aug 19;6(8):e23767. doi: 10.1371/journal.pone.0023767 (PMC3158794; doi:10.1371/journal.pone.0023767)
Supplement: Supplementary file 3 [file pone.0023767.s003.doc]

Table S1. Host selection ratios for *Culex pipiens* collected in 11 field sites in southwest suburban Chicago, Illinois, 2005-2008. Includes avian species that were not observed in blood meals at a site (given a value of one) and those that were not recorded during avian surveys (given value of lowest observed bird density).

	Site 1	Site 3	Site 5	Site 7	Site 10	Site 11	Site 12	Site 15	Site EC	Site SC	Site WW
Host[a]	$\hat{w}_i$	$\hat{w}_i$	$\hat{w}_i$	$\hat{w}_i$	$\hat{w}_i$	$\hat{w}_i$	$\hat{w}_i$	$\hat{w}_i$	$\hat{w}_i$	$\hat{w}_i$	$\hat{w}_i$
American Crow				_2.85_						_4.83_	
American Goldfinch	_2.30_	_4.84_	_1.32_		_3.37_	_0.91_	_0.84_		_1.84_	1.20	_0.92_
American Kestrel		_282.16_									
American Robin	0.94	1.07		1.96	2.00	0.97	10.13	1.25	1.12	0.32*	0.85
Baltimore Oriole			_42.11_			_34.94_		_43.54_		_34.40_	_52.85_
Black-capped Chickadee	_5.98_						_49.18_	_4.43_	_3.59_	_14.00_	
Brown-headed Cowbird	_10.94_		_10.45_	_10.09_				_2.70_	_2.19_	_0.95_	_13.12_
Blue Jay	_10.23_	_5.37_	_4.89_	18.87			4.99		_2.73_	_3.99_	12.27
Brown Thrasher										_65.56_	
Cedar Waxwing	_2.70_					_106.54_		_1.33_	_1.08_	1.27	
Chipping Sparrow	_3.24_	_10.20_	_9.28_	_2.24_		1.93		_0.96_	_1.94_	_0.54_	
Common Grackle	_2.27_	_0.95_	_0.29_	_0.84_	_0.67_	_0.51_	_0.08*_	_1.12_	_0.23_	_0.89_	_0.91_
Cooper's Hawk					_98.39_						
Common Yellowthroat										_2.60_	
Downy Woodpecker				_15.72_				_5.61_		_6.65_	_6.81_
Eastern Kingbird					_11.36_					_4.04_	
European Starling	3.88	0.51	3.24	0.48	_0.38_	0.43	0.12*	_0.79_		_0.25_	1.12
Gray Catbird	_6.62_					_2.63_		_6.54_	_6.82_	2.07	_0.57_
Hairy Woodpecker									_0.10*_		
House Finch	2.29	4.81	4.38	33.81	3.35	25.42	6.71	2.01		14.30	32.95
House Sparrow	0.14*	0.51	0.38*	0.45	0.17*	0.29*	0.13*	0.07*	1.77	0.78	0.30
House Wren	_9.77_	_10.26_	_18.67_			_5.16_	49.18	3.86	_19.62_	7.62	
Indigo Bunting						_37.42_				_12.28_	_56.58_
Killdeer									_0.96_		_4.20_
Mallard											_0.61_
Mourning Dove	1.70	1.54	1.15	0.34	0.54	2.10	0.38	_0.40_	2.61	3.44	2.64
Monk Parakeet							_0.03*_		_28.03_		
Northern Cardinal	1.93	12.18	9.98	6.42	_1.42_	4.60	1.42	8.92	2.35	_2.26_	3.71
Red-eyed Vireo		_28.16_					_9.82_				_32.17_
Ring-necked Pheasant										_45.80_	
Rock Pigeon						_0.32_		_0.89_			
Red-winged Blackbird						_0.46_			_0.26_	_0.18*_	_0.18*_
Scarlet Tanager							_49.18_				
Song Sparrow		_141.08_				_4.51_		_16.85_	_0.84_	_0.63_	_1.57_
Swamp Sparrow	_134.34_					_106.54_					
Veery									_2.07_		
Warbling Vireo									_13.65_	_9.28_	_1.43_
Willow Flycatcher									_67.21_	_1.71_	
Northern Flicker							_49.18_		_9.52_	_13.23_	
Yellow Warbler										_29.31_	
Sample size	18	27	31	29	23	43	158	21	35	19	23

[a]Scientific names can be found by accessing the American Ornithologists' Union Check-list (http://www.aou.org/checklist/north/index.php).

[b]Italicized host selection ratios imply that a blood meal of one or a relative density of the lowest observed bird was assigned.

*Statistically significant non-random host selection at $P < 0.05$.
