# Supplementary material for: Fine-Scale Variation in Vector Host Use and Force of Infection Drive Localized Patterns of West Nile Virus Transmission
Source: PLoS One. 2011 Aug 19;6(8):e23767. doi: 10.1371/journal.pone.0023767 (PMC3158794; doi:10.1371/journal.pone.0023767)
Supplement: Supplementary file 4 [file pone.0023767.s004.doc]

Table S2. Number of infectious *Cx. pipiens* (i.e. force of infection) resulting from feeding on different avian hosts by site, suburban Chicago, Illinois, 2005-2008. Number of infectious mosquitoes were calculated by $B_i^2 * C_i$, where B is the proportion of blood meals from host species i and C is the host competence index. All sites combined includes additional sites with samples sizes of < 20 *Cx. pipiens* blood meals. Species are ranked based on force of infection values from highest to lowest for all sites combined.

		1	3	5	7	10	11	12	15	EC	SC	WW	All sites
Host species[a]	C_i	$B_i^2 * C_i$	$B_i^2 * C_i$	$B_i^2 * C_i$	$B_i^2 * C_i$	$B_i^2 * C_i$	$B_i^2 * C_i$	$B_i^2 * C_i$	$B_i^2 * C_i$	$B_i^2 * C_i$	$B_i^2 * C_i$	$B_i^2 * C_i$	$B_i^2 * C_i$
American Robin	1.04	0.084	0.019	0.030	0.036	0.332	0.037	0.573	0.132	0.507	0.045	0.051	0.160
House Sparrow	1.23	0.011	0.109	0.073	0.081	0.005	0.028	0.004	0.002	0.001	0.002	0.009	0.017
House Finch	1.28	0.003	0.004	0.001	0.011	0.006	0.022	0.001	0.007	0.000	0.009	0.009	0.004
Northern Cardinal	0.38	0.001	0.000	0.018	0.002	0.000	0.002	0.000	0.024	0.000	0.000	0.005	0.002
Blue Jay	2.38	0.000	0.000	0.000	0.012	0.000	0.000	0.001	0.000	0.000	0.000	0.007	0.001
Mourning Dove	0.09	0.005	0.001	0.000	0.000	0.000	0.005	0.000	0.000	0.001	0.004	0.000	0.001
European Starling	0.17	0.002	0.000	0.000	0.000	0.000	0.000	0.000	0.000	0.000	0.000	0.001	5.78E-05
American Kestrel	0.78	0.000	0.002	0.000	0.000	0.000	0.000	0.000	0.000	0.000	0.000	0.000	1.65E-05
House Wren	0.77	0.000	0.000	0.000	0.000	0.000	0.000	0.000	0.001	0.000	0.001	0.000	1.64E-05
Scarlet Tanager	0.77	0.000	0.000	0.000	0.000	0.000	0.000	0.000	0.001	0.000	0.000	0.000	1.64E-05
Cedar Waxwing	0.77	0.000	0.000	0.000	0.000	0.000	0.000	0.000	0.000	0.000	0.001	0.000	1.64E-05
Common Grackle	1.40	0.000	0.000	0.000	0.000	0.000	0.000	0.000	0.000	0.000	0.000	0.000	1.32E-05
Chipping Sparrow	0.97	0.000	0.000	0.000	0.000	0.000	0.000	0.000	0.000	0.000	0.000	0.000	9.14E-06
Song Sparrow	0.97	0.000	0.001	0.000	0.000	0.000	0.000	0.000	0.000	0.000	0.000	0.000	9.14E-06
Black-capped Chickadee	0.77	0.000	0.000	0.000	0.000	0.000	0.000	0.000	0.000	0.000	0.000	0.000	7.27E-06
Red-winged Blackbird	0.42	0.000	0.000	0.000	0.000	0.000	0.000	0.000	0.000	0.000	0.000	0.000	4.00E-06
Common Canary	1.28	0.000	0.000	0.000	0.000	0.000	0.000	0.000	0.000	0.000	0.000	0.000	3.01E-06
American Goldfinch	1.28	0.000	0.000	0.000	0.000	0.000	0.000	0.000	0.000	0.000	0.002	0.000	3.01E-06
Cooper's Hawk	0.78	0.000	0.000	0.000	0.000	0.001	0.000	0.000	0.000	0.000	0.000	0.000	1.84E-06
Swainson's Thrush	0.12	0.000	0.000	0.000	0.000	0.000	0.000	0.000	0.000	0.000	0.000	0.000	1.11E-06
Veery	0.39	0.000	0.000	0.000	0.000	0.000	0.000	0.000	0.000	0.000	0.000	0.000	9.08E-07
Gray Catbird	0.08	0.000	0.000	0.000	0.000	0.000	0.000	0.000	0.000	0.000	0.001	0.000	7.34E-07
Brown Thrasher	0.19	0.000	0.000	0.000	0.000	0.000	0.000	0.000	0.000	0.000	0.000	0.000	4.38E-07
Northern Flicker	0.14	0.000	0.000	0.000	0.000	0.000	0.000	0.000	0.000	0.000	0.000	0.000	3.35E-07
Rock Pigeon	0.00	0.000	0.000	0.000	0.000	0.000	0.000	0.000	0.000	0.000	0.000	0.000	0.00E+00
Sample size		21	37	41	43	30	53	168	28	40	24	36	652

[a]Scientific names can be found by accessing the American Ornithologists' Union Check-list (http://www.aou.org/checklist/north/index.php).
